# Elastic wave prospecting of water-conducting fractured zones in coal mining

**DOI:** 10.1038/s41598-024-57557-2

**Published:** 2024-03-25

**Authors:** Bingchao Zhao, Shenglin He, Kun Bai, Xiaoxiao Lu, Wei Wang

**Affiliations:** 1 Xi’an University of Science and Technology, Xi’an, 710054 Shaanxi China; 2 Shaanxi Xiaobaodang Mining Company, Yulin, 719000 Shaanxi China; 3 Shaanxi Caojiatan Mining Company, Yulin, 719000 Shaanxi China

**Keywords:** Mining engineering, Coal seam, Development of water-conducting fractures, Microseismic monitoring, Seismic exploration, Civil engineering, Engineering

## Abstract

In order to understand the development law of water-conducting fractures in overlying strata during the mining process of coal seam, an elastic wave exploration method based on key stratum theory is proposed to predict the height of water-conducting fracture zone. Taking Yushen mining area as the background, the development and evolution of fractures and the three-dimensional distribution characteristics of water-conducting fracture zone are studied by combining well-ground microseismic monitoring, high-density three-dimensional seismic exploration, borehole investigation, FLAC3D numerical simulation and similar physical simulation tests. The results indicate that the trial mining face's fracture-to-coal ratio ranges from 25.86 to 30.76, with the maximum fracture-to-coal ratio near the cutting eye at 30.76 and the minimum in the central portion of the trial mining face at 25.86. The primary characteristics of rock mass fracture distribution in the mined area are the development of fractures predominantly along high-angle and even vertical bedding planes. Within the fracture zone, fractures increase from top to bottom, with high-angle fractures developing in the lower section and high-angle and horizontal fractures developing simultaneously in the upper section. The water-conducting fracture zone undergoes a developmental process from inception to development, reaching its maximum height, and eventually stabilizing as coal seam mining progresses, overlying rock subsides, strata separation, and damage formation. The three-dimensional shape of the water-conducting fracture zone in the roof of the Yushen mining area exhibits a morphological pattern where the height of the fracture zone gradually decreases from the cutting eye towards the goaf. It also transitions from high to low along both sides and from the periphery towards the interior of the working face. In the trend and strike directions, it exhibits saddle-like characteristics. By comparing the monitoring results, the rationality of the elastic wave prospecting method for predicting the height of water-conducting fracture zones based on critical layer theory was verified. This research holds significant reference value for coal mining under similar geological conditions, especially in terms of water preservation during mining operations.

## Introduction

Coal mining can cause changes in geological structures and conditions, leading to damage to overlying strata and surface disturbances, which in turn can trigger a series of geological environmental issues, such as groundwater loss and land desertification. The accurate detection of the development characteristics of water-conducting fracture zones holds significant importance for determining the type of roof water hazards, establishing water-protective coal mining zones, and preventing roof water hazard accidents^1–3^.

The main methods for investigating water-conducting fracture zones include theoretical calculations, empirical formulas, similar material simulations, numerical simulations, and field measurements^4–6^. The prediction of water-conducting fracture zone heights is a crucial aspect of coal mining water management, and accurate predictions are essential for safe mining. The empirical formulas in the "Three Down Regulations" are primarily derived from regression analysis of measured data for thin and medium-thick coal seam water-conducting fracture zone heights in the early North China region. However, these formulas only consider mining height as the influencing factor and exhibit significant predictive errors under different mining conditions, lacking strong guidance. These empirical formulas only consider the coal seam thickness parameter, without direct consideration of the impact of overlying rock lithological structures^7^. While neural network methods consider multiple influencing factors, they are prone to finding local optima, and their accuracy is low with few samples and overfitting with many samples, leading to low generalization ability. Currently, there are issues with the accuracy of water-conducting fracture zone height prediction models^8,9^. Physical similarity simulations and numerical simulations are unable to accurately simulate complex geological conditions and stress states, making them suitable for qualitative research on overlying rock strata destruction patterns but less suitable for quantitative prediction of water-conducting fracture zone heights^10^.

Field measurements are considered the most reliable method^11–13^. Currently, both domestic and international methods for field detection of water-conducting fracture zones include borehole flushing fluid observation, underground borehole water injection, borehole television, and other techniques. However, these methods are costly, and the results are only discrete borehole data, which may introduce errors in interpolation between boreholes^14^. Borehole observation methods are limited by cost, and the small number of boreholes may result in significant monitoring errors due to the change in stress state when rock cores are taken from the boreholes. In recent years, microseismic or seismic monitoring techniques, as a form of three-dimensional spatial monitoring technology for rock mass micro-cracking, have rapidly developed and found significant applications in coal mine safety production^15–17^. Microseismic monitoring is cost-effective, has short monitoring intervals, and provides continuous dynamic monitoring results. Three-dimensional seismic technology offers continuous data and high vertical resolution, partially compensating for the limitations of borehole data. Therefore, the comprehensive use of elastic wave detection methods and multi-data fusion analysis, mutually complementing and confirming each other, is a necessary means to investigate water-conducting fracture zones.

The evolution of water-conducting fractures induced by coal seam mining is related to the breaking movement of the overlying rock layers. The breaking movement of overlying rock layers is controlled by key layers, and the evolution of water-conducting fractures is inevitably influenced by the structure of these key layers^18–20^. Based on the theory of key layers, an elastic wave exploration method for water-conducting fracture zone height can fully reflect the structural characteristics of key layers in specific mining conditions, avoiding the statistical homogenization of overlying rock lithology and reducing monitoring errors. In summary, this paper derives a formula for calculating water-conducting fracture zone height under coal seam conditions in the Yushen mining area based on the theory of key layers and establishes an elastic wave exploration method for water-conducting fracture zone height. Taking the 112201 working face of Xiaobaodang No. 1 coal mine as the research object, the study investigates the development height and three-dimensional spatial characteristics of water-conducting fracture zones through theoretical analysis, numerical simulations, similar simulations, and underground field measurements. The research findings provide essential reference information for water-protective coal mining and safe mining in similar mines in the Yushen mining area of China.

## High-elasticity wave prospecting method for water-conducting fracture zones based on key layer theory

### Theoretical principle

According to the key layer theory, when a key layer experiences a rupture, the simultaneous bending and subsidence of the upper rock layers or some rock layers occur. Therefore, in determining the height range of the development of water-conducting fracture zones, one can analyze the rupture conditions of the key layer^21–23^. The key layer's rupture conditions can be determined by comparing the maximum deflection of the key layer during coal seam mining with the maximum free space below the key layer. When the maximum deflection of the key layer is less than the maximum free space below the key layer, the rock layers experience failure, and the water-conducting fracture zone continues to develop. The maximum deflection and maximum free space for each key layer throughout the coal seam mining process are calculated using Eq. (1).

1 $$f_{i\max } > f = m - \sum\limits_{x = 1}^{i - 1} {h_{x} } \left( {k_{x} - 1} \right)$$

where *m* is the mining thickness, m; *h*_*x*_ is the thickness of the lower layer *x* of rock layer, m; *k*_*x*_ is the residual fragmentation coefficient of the lower *x* of rock layer; *f*_*imax*_ is the maximum deflection of the *i* layer of rock layer.

When the key layer is a solid support beam, the maximum deflection is given by:

2 $$f_{\max } = \frac{{ql^{4} }}{384EI}$$

where *q* is the load supported by the key layer in N/m^2^; *l* is the length of the rock beam affected by mining, m; *E* is the elastic modulus of the rock beam, Pa. *I* is the moment of inertia of the rock beam's cross-section, and *I* = 1/12*bh*^3^; *b* is the width of the rock beam, m; *h* is the thickness of the rock beam, m.

Practical observations show that when the advancing length and width of the working face both reach 1.2–1.4*H* (where *H* is the depth of the coal seam), full mining influence is achieved at the surface. For the overlying rock layers, when the advancing length and width of the working face reach 1.2–1.4*h* (where *h* is the distance from the rock layer to the coal seam roof), the overlying rock layers reach full mining influence. When the advancing length of the working face is 2*r*(*z*), the length of the rock beam affected by mining is 4*r*(*z*). When the advancing length and width of the working face exceed 1.4*h*, the overlying rock layers are under super-sufficient mining conditions, resulting in downward bending and maximum subsidence of the intermediate flat portion of the rock beam, with zero curvature deformation. When the advancing length of the working face is greater than 2*r*(*z*), the range of the rock beam affected by mining is greater than 4*r*(*z*), and since the middle flat portion of the rock beam has zero curvature, it can be approximately considered that the range affected by mining in the overlying rock beam is 4*r*(*z*).

When the key layer ruptures, the bending and subsidence of the upper rock layers or part of the rock layers occur simultaneously. Therefore, to determine the development height of water-conducting fracture zones, you can analyze the rupture conditions of the key layer. The development height of the upward water-conducting fracture zone can be predicted based on the rupture conditions of the key layer. The process for determining the maximum development height range of the upward water-conducting fracture zone is as follows: when the main key layer has not ruptured, determine the rupture conditions of the first sub-key layer beneath the main key layer, and continue this process until the first sub-key layer that has ruptured is identified. The development height range of the water-conducting fracture zone is between the ruptured first sub-key layer and the unruptured key layer. If the main key layer ruptures, the water-conducting fracture zone develops to the upper part of the fracture zone and the lower part of the bending subsidence zone. If no bending subsidence zone exists in the overlying rock, the water-conducting fracture zone develops to the surface.

In the case of full collapse mining, the relationship between the limit curvature *K* of the top rock layer of the water-conducting fracture zone and the upward development height *h*_*s*_ is given by Eq. (3):

3 $$h_{s}^{2} = \frac{{7.25w_{i} }}{K(\cot \delta + \cot \varphi )}$$

where *w*_*i*_ is the subsidence value of the topsoil layer of the water-conducting fracture zone; *m* is the mining thickness; *δ* is the angle of rock layer movement; *φ* is the angle of full mining.

The angle of full mining refers to the angle formed between the edge points of the flat bottom of the depression basin profile under full mining influence and the mining boundary line and the coal seam.

4 $$\cot \varphi = \frac{L}{{h^{\prime} }}$$

where *L* is the horizontal distance from the edge points of the depression basin to the mining boundary, m; *h'* is the vertical distance from the edge points of the depression basin to the coal seam being mined, m.

The calculation formula for the angle of full mining and the angle of rock layer movement is given by Eq. (5):

5 $$\cot \delta = \frac{r(z)}{h}$$

where *h* is the distance from the rock layer to the coal seam roof, m; *r*(*z*) is the radius of influence of the coal seam being mined in the rock layer, m.

The relationship between the limit curvature of the top rock layer of the water-conducting fracture zone and the load on the upper part of the rock layer is given by Eq. (6):

6 $$K^{\prime}_{\max } = \frac{{ql^{2} }}{12EI}$$

where *q* is the load on the hard rock layer, N; *l* is the length of the rock beam, m; *E* is the elastic modulus of the rock beam, Pa; *I* is the moment of inertia of the rock beam's cross-section, m^4^.

7 $$I = \frac{1}{12}bh^{3}$$

where *b* is the width of the rock beam, m; *h* is the thickness of the rock beam, m.

During the coal seam mining process, the overlying soil layers gradually experience bending deformation due to the influence of mining. When the working face advances a relatively short distance, the impact of mining on the surface is minor, and the horizontal deformation of the surface soil layers does not reach the limit tensile deformation value. During this stage, downward fractures generally do not appear on the surface.

As the working face progressively advances, the surface or loose layers undergo tensile deformation, leading to the gradual development of downward fractures. While the downward water-conducting fracture zone is evolving, when the tensile deformation of a certain layer, which acts as an aquitard, exceeds its maximum tensile deformation value, that aquitard layer will fail, leading to the continued downward development of the water-conducting fracture zone. Therefore, assessing the tensile deformation of the remaining thickness of the aquitard layer is crucial in determining the depth of development of the downward water-conducting fracture zone.

To determine the tensile deformation status of the remaining thickness of the aquitard layer, you can make preliminary assessments based on the location of the aquitard layer. If the aquitard layer is in the caving zone, it will fully collapse. If it's in the fracture zone, it will experience varying degrees of collapse. If it's in the bending subsidence zone, it's considered to have minimal damage and is presumed not to have fully collapsed. Therefore, the assessment of the depth of development of the downward water-conducting fracture zone primarily focuses on the bending subsidence zone.

The relationship between the depth of development *h*_*x*_ of the downward fracture and the maximum tensile deformation *ɛ* can be expressed as:

8 $$h_{x} = h_{g} - 2\rho \varepsilon$$

where *ρ* represents the curvature radius of the aquitard's neutral layer, m; The relationship between curvature radius and curvature *K* is given by *ρ* = 1/*K*; *h*_*g*_ stands for the thickness of the aquitard, m.

The calculation of the maximum surface curvature *K*_*max*_ can be determined using the probability integral method and is expressed by:

9 $$K_{\max } = \frac{{1.52w_{\max } }}{{r^{2} }}$$

where the influence radius *r* of the surface due to mining activities can be derived based on the coal seam depth *H* and the tangent value of the main influence angle tan*β* using the equation:

10 $$r = \frac{H}{\tan \beta }$$

The relationship between the depth of the downward fractures *h*_*x*_ and the horizontal deformation value of the surface *ɛ* as follows:

11 $$h_{x} = h_{g} - 1.316\frac{{H^{2} \varepsilon }}{{w_{\max } (\tan \beta )^{2} }}$$

In the course of coal seam mining, the subsidence of the surface can be estimated through either similar simulation experiments or the establishment of mobile observation stations on the surface. Laboratory simulation experiments and field measurements have shown that cracks tend to appear when the horizontal tensile deformation of common soil layers reaches 1–2 mm/m. However, in areas with thick sand layers covering the surface, horizontal tensile stresses are more easily released, making it less likely for cracks to develop. Taking the minimum critical value for tensile failure as 6 mm/m, the appearance of surface cracks is anticipated when the horizontal deformation of the sand layer reaches 6 mm/m. Considering the self-healing capacity of the surface ecosystem, these cracks tend to gradually repair over time, particularly when coal seams are mined individually.

### Detection method

Most microseismic events occur on the cross-sections of fractures in coal rock layers, and rock acoustic emission and microseismic monitoring techniques are used to assess the stability of coal rock formations by detecting the energy released in the form of elastic waves during fracture propagation^24^. Cai and Kaiser^25^ categorized microseismic events based on their frequency, considering phenomena like acoustic emissions, microseismic activity, rock bursts, and earthquakes as vibration events with different oscillation frequencies. The fracturing events within rocks in the Earth's interior are generally referred to as earthquakes. Depending on the scale of these fracturing events, they can be classified as microseismic events, small earthquakes, moderate earthquakes, strong earthquakes, major earthquakes, or giant earthquakes. Based on their causes, they can be categorized as collapse-induced earthquakes, volcanic earthquakes, human-induced earthquakes (e.g., mining-induced seismicity, fluid injection-induced earthquakes), and tectonic earthquakes. It's worth noting that there are first-order statistical similarities in the frequency-magnitude relationships and the spatial–temporal distribution of deformation for fractures at various scales, ranging from millimeter-scale fractures in rock samples under load to meter-scale mining-induced microseismic activity and kilometer-scale earthquakes. Creep tests on rock samples at different scales reveal typical characteristics with three phases: initial, steady, and accelerating creep. Major fractures or macroscopic failure typically result from the nucleation, propagation, and connection of microcracks^26^. Vallianatos^27^ and others have also indicated that the fracture behavior in rock (bodies) from laboratory scale to the Earth's scale might exhibit universal patterns. The real-time monitoring capabilities of microseismic techniques complement the high-resolution imaging provided by three-dimensional seismic exploration, allowing for the timely identification and mitigation of potential hazards.

The overlying rock strata of coal seams exhibit a layered structure with variations in thickness and rock strength. Consequently, the deformation and damage of overlying rock strata differ between layers, giving rise to zonation in the overlying rock strata of coal seams. In a given zone, the rock typically exhibits a predominant deformation or damage state and is accompanied by other forms of deformation or states. The extent to which this zoning is apparent depends primarily on the mechanical disparities between the rock layers. In essence, the theory of zonation in overlying rock strata is closely related to the methods for measuring deformation, damage, and stress in the overlying rock strata. When mining depths are substantial, there might be multiple layers of relatively hard overlying rock strata. Among these, the layer that has the most significant influence on the deformation and damage of the entire rock mass is termed the "primary key layer," while the layer that has a greater influence on the deformation and damage of local rock strata is called a "secondary key layer." The zonation in overlying rock strata implies that the non-uniformity of mechanical parameters along the vertical direction of the rock mass leads to the division of the overlying rock strata into zones. The rock mass on either side of the key layer exhibits significant differences in terms of deformation, stress, and damage. These differences inevitably result in variations in the spatial and energy distribution of microseismic events. Therefore, the differential spatial and energy distribution of seismic events can be used to locate the key layer.

Characteristics of rock deformation and damage in different zones:

1. Collapse zone: In the collapse zone, rock mass directly collapses into the mined-out area as mining progresses. This zone is characterized by rock fractures and faults.
2. Rock block area: In this area, the layered rock mass fractures into blocks, but these blocks do not collapse; instead, they are interconnected, forming rock beams.
3. Vertical fracture zone: In this area, the layered structure of the rock mass remains relatively intact, but vertical fractures develop, penetrating the entire rock layer.
4. Vertical fracture zone with interbedding: In this area, the rock mass exhibits both vertical fractures and interbedding phenomena. However, the extent of interbedding is minimal, with vertical fractures being the primary feature, though not fully connected.
5. Interbedding zone: In this area, the rock mass exhibits few vertical fractures and primarily shows horizontal interbedding.
6. Bend and subsidence zone: In this area, there are neither vertical fractures nor horizontal interbedding, instead, the rock layers exhibit coordinated deformation.

**Figure 1:**
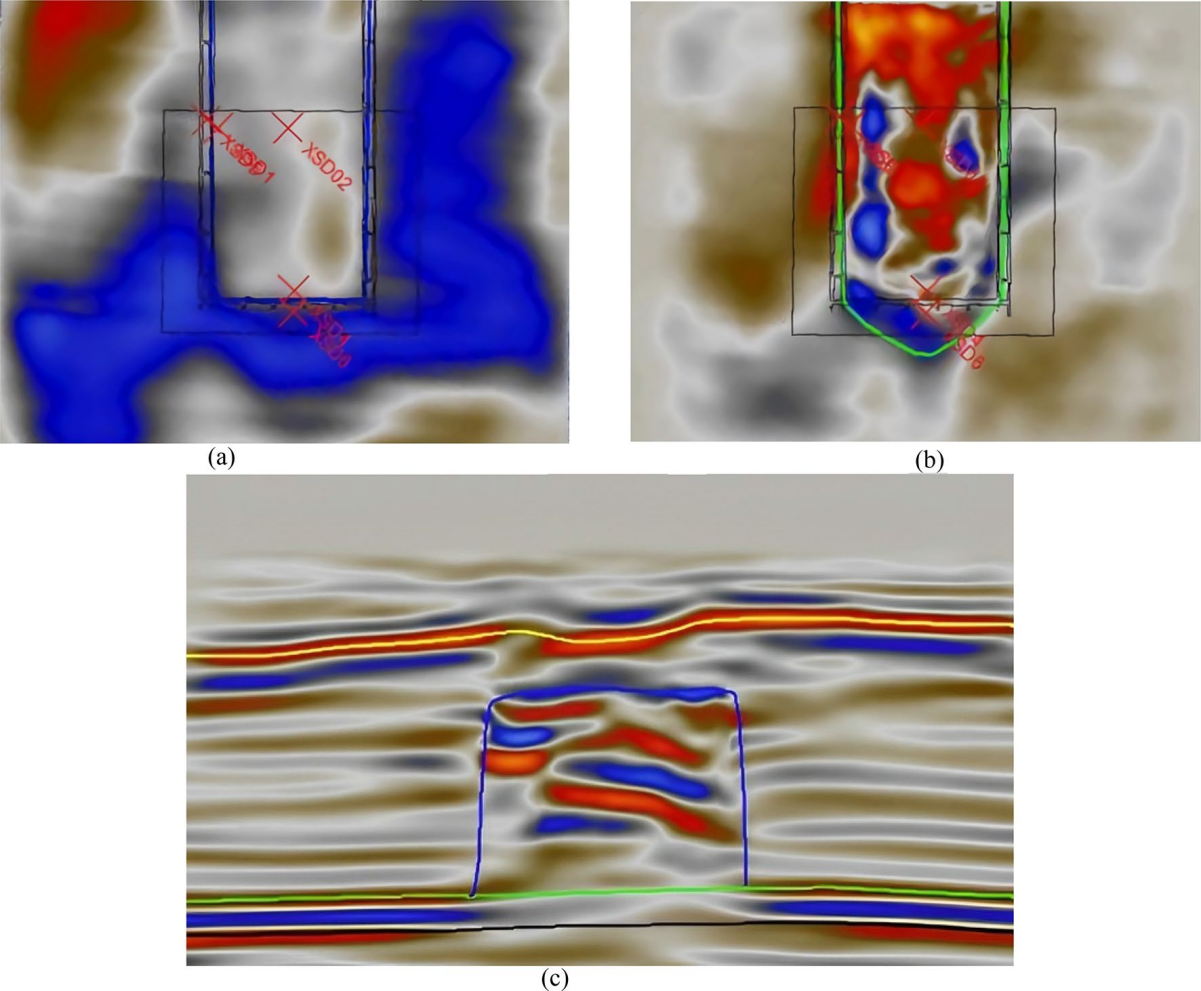
3D seismic time profiles. (**a**) Horizontal cross-section of the collapse zone. (**b**) Horizontal cross-section of the fracture zone. (**c**) Seismic time profile of the water-conducting fracture zone.

The collapse zone occurs in the upper rock layers directly above the mined-out area, where the strata are fractured and the geological properties change significantly, leading to the disruption of wave groups and disordered waveforms in seismic records (Fig. 1a). The fracture zone, theoretically, corresponds to the area where the strata are not completely disrupted, but fractures of various sizes occur, affecting the continuity of strata and the energy of seismic reflection waves (Fig. 1b). The bend and subsidence zone involves ductile deformation, resulting in good continuity and energy of wave groups, but minor fissures and cracks develop inside the strata, leading to clear strata anisotropy.

**Figure 2:**
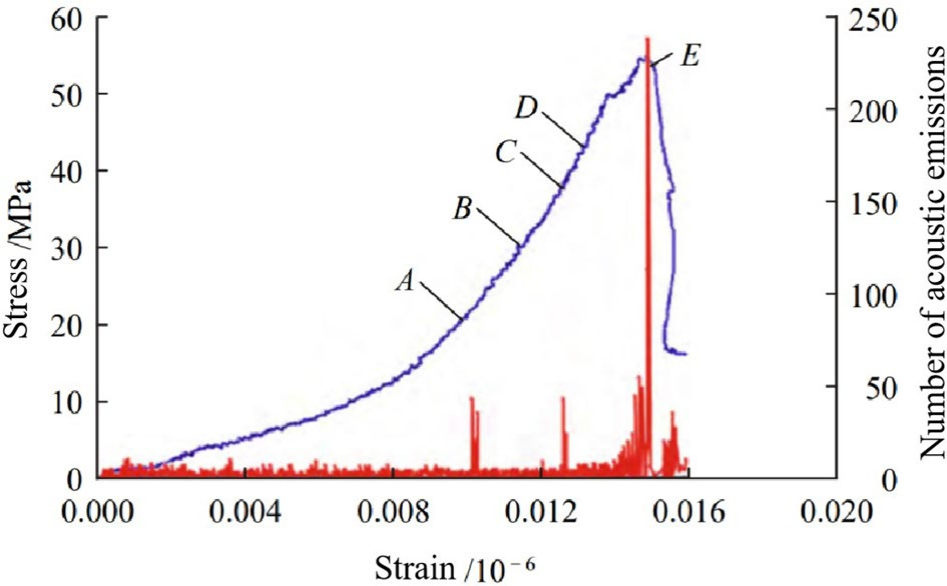
Relationship between the full stress–strain curve of rock fracture process and acoustic emission counts^28^.

Tang et al.^28^ conducted research on the relationship between the full stress–strain curve of rock and the number and energy of acoustic emission events. They suggested that both acoustic emissions and microseismic fracturing are closely related to the stress and deformation of the rock mass. The stress–strain curve of rock is divided into five stages: rock sample compaction, elastic deformation, stable crack propagation, unstable crack propagation, and rock sample failure (Fig. 2). Different stages correspond to different rock deformation, loss, and damage conditions, with distinct acoustic emission characteristics. During the rock sample compaction stage (Stages O to A), very few acoustic emission events occur. In the elastic deformation stage (Stages A to C), acoustic emission events are infrequent and have relatively low amplitudes. The stable crack propagation stage (Stages C to D) is marked by progressively more frequent acoustic emission activity with increasing amplitudes and frequencies. In the unstable crack propagation stage (Stages D to E), there is a sharp increase in acoustic emission events, characterized by significant amplitude fluctuations and higher energy release. Beyond Point E, in the rock sample failure stage, acoustic emission events are not well captured, mainly due to the limitations of the pressure machine's rigidity. Since the rock deformation and damage resulting from mining often span a wide range of scales, covering all stages of the stress–strain curve, the rock exhibits diverse characteristics of stress and strain, with seismic events representing various deformation stages. Therefore, it is feasible to determine the zonation of coal mine roof strata by studying the spatial and temporal distribution patterns of microseismic information in rock at different stages of the stress–strain curve. Rock at an engineering scale typically produces a wide range of microseismic event energy and frequency from the initiation of microcracks to the unstable crack propagation stage, which encompasses Stages B to E. It's important to note that monitoring all microseismic events is often challenging and unnecessary. Precursor information from micro-cracking before the rock fractures is particularly significant for hazard early warning. Consequently, when designing microseismic monitoring schemes, the focus is often on monitoring from the initiation of microcracks to the stage of unstable crack propagation (Stages B to E).

The vertical zonation model of the coal mine roof based on microseismic monitoring corresponds to the traditional three-zone model to a certain extent: the collapse zone in the model based on microseismic monitoring corresponds to the collapse zone in the traditional three-zone model. Since the traditional three-zone model is based on the formation of through-going fractures, the rock block area and the vertical fracture penetration zone in the vertical zonation model of coal mine roof based on microseismic monitoring correspond to the fracture zone in the three-zone model. The vertical fracture zone, interbedding zone, and bend and subsidence zone in the vertical zonation model based on microseismic monitoring correspond to the bend and subsidence zone in the three-zone model. The rock mass in the collapse zone is expected to be in the rock sample failure stage, while the rock mass in the fracture zone is likely in the unstable crack propagation stage, and the rock mass in the bend and subsidence zone can be in any stage from rock sample compaction, elastic deformation, to stable crack propagation. Because microseismic monitoring primarily targets the period from the initiation of microcracks to the unstable crack propagation stage (Stages B to E), it's possible to use microseismic monitoring to observe the collapse zone and fracture zone, but it may not fully capture the bend and subsidence zone. However, it can detect the boundary between the bend and subsidence zone and the fracture zone, thus enabling the determination of the bend and subsidence zone. By studying the spatial and energy distribution differences of seismic events, the feasibility of locating the key layer is evident. The key layer is typically the boundary between the two zonation areas of the roof. Therefore, locating the key layer is the central idea behind zonation of the coal mine roof based on microseismic monitoring. Since microseismic monitoring data are statistical in nature, accurate location of the key layer requires comprehensive analysis based on the differential distribution of microseismic events and energy along both sides of the key layer, in conjunction with geological information (primarily borehole logs)^29,30^.

## Application of elastic wave prospecting method based on key layer theory

### Overview of the coal mine site

The Yushen mining area is situated in the central part of the Jurassic coalfield in northern Shaanxi. It boasts favorable coal seam conditions, abundant reserves, simple geological structures, excellent coal quality, and low levels of harmful components, earning it the reputation of being an "eco-friendly coal." The construction and production in this mining area are of great significance for the development of China's national economy. Simultaneously, the mining area is located in the Maowusu Desert region, characterized by fragile ecological conditions and water scarcity.

Large-scale coal extraction can disrupt and deform the overlying rock and soil layers, jeopardizing the existing conditions of underground water resources, causing fluctuations in the groundwater level, and potentially leading to adverse environmental impacts in the region. Therefore, while developing coal resources, it is essential to protect underground water resources, maintain ecological water levels, safeguard the local environment, and promote the sustainable development of the regional ecological environment. This is a challenging yet crucial task faced during the coal resource development process in the Yushen mining area.

Xiaobaodang Wellfield No. 1 is located in the central part of the third-phase planning area of the Yushen mining area. It is a large, modern mine with an annual production capacity of tens of millions of tons, invested in and built by Shaanxi Xiaobaodang Mining Co., Ltd. The No. 1 well has a designed production scale of 15 million tons per year, with a service life of 73.8 years. It uses inclined shaft development and includes four shafts: the main inclined shaft, auxiliary inclined shaft, and intake and return air shafts. Currently, Well No. 1 is undergoing trial mining at the 112201 working face.

As shown in Table 1, which presents the K4-3 borehole comprehensive columnar chart and the rock mechanics parameters, the identification of key layers in the 112201 working face is as follows: there are four key layers in the 112201 working face. The first sub-key layer is a medium-grain sandstone located 3.37 m from the top of the coal seam, with a thickness of 35.29 m. The second sub-key layer is a fine-grain sandstone situated 50.97 m from the top of the coal seam, with a thickness of 13.5 m. The third sub-key layer is a medium-grain sandstone located 79.17 m from the top of the coal seam, with a thickness of 19.35 m. The main key layer is a fine-grain sandstone situated 113.66 m from the top of the coal seam, with a thickness of 30.85 m.

**Table 1:** Physical–mechanical parameters of the roof rocks for the 112201 working face.

Layer no	Thickness (m)	Rock type	Natural density (KN/m^3^)	Tensile strength (MPa)	Elastic modulus (GPa)	Cohesion (MPa)	Internal friction angle (°)
1	6.8	Fine sand	1.45	0.1	0.1		
2	83.2	Red clay	1.55	0.1	0.1	0.03	25.8
3	11.77	Siltstone	23.6	1.02	19.58	2.44	38.75
4	5.43	Fine-grained sandstone	23.2	1.26	25.47	2.88	38.84
5–6	21.2	Siltstone	23.2	1.1	21.24	2.34	38.69
7	9.7	Fine-grained sandstone	22.9	1.25	36.8	3.82	36.97
8	5.98	Sandy mudstone	23.7	1.62	30.27	3.19	37.87
9	13.21	Siltstone	24.1	1.25	23.25	2.44	38.75
10	8.5	Fine-grained sandstone	22.9	1.96	36.58	3.82	36.97
11	3.6	Sandy mudstone	23.7	1.62	30.27	3.19	37.87
12	10.9	Fine-grained sandstone	22.9	1.96	36.58	3.82	36.97
13	25.95	Siltstone	23.5	1.32	24.32	2.67	37.93
14	30.85	Fine-grained sandstone	23.1	1.5	26.57	2.88	38.84
15	16.91	Siltstone	23.6	0.98	16.58	1.94	39.29
16	6.1	Sandy mudstone	23.7	1.62	30.27	3.19	37.87
17	8.89	Fine-grained sandstone	23.2	1.4	27.5	2.8	37.42
18	19.35	Medium-grained sandstone	22.8	1.75	32.45	3.49	38.35
19	9.05	Fine-grained sandstone	23.2	1.4	27.45	2.28	37.42
20	0.65	1-1 coal	13.2	0.56	8.58	0.92	35.96
21	3.25	Siltstone	23.5	1.34	24.68	2.27	37.42
22	0.55	Coal	13.2	0.56	10.12	0.92	35.96
23	1.2	Siltstone	23.9	1.2	21.47	3.53	38.16
24	13.5	Fine-grained sandstone	2.31	2.21	41.2	4.49	38.35
25	1.4	Fine-grained sandstone	22.1	1.85	31.27	3.59	37.11
26	10.71	Siltstone	23.9	1.23	26.5	2.14	39.85
27	35.29	Medium-grained sandstone	23.1	1.98	31.07	3.29	39.17
28	3.37	Fine-grained sandstone	23.3	1.65	29.87	3.12	37.35
29	5.78	2-2 coal	13.1	0.48	7.54	0.61	38.48

### High-density 3D seismic prospecting for water-conducting fracture zones

#### High-density 3D seismic prospecting system

For this high-density 3D seismic prospecting, a 16-line, 4-source point grid system was employed with a receiver line spacing of 40 m and an offset distance of 10 m. Given the coal seam's structural characteristics in the area, a midpoint-triggered observation system was used along the vertical direction. The data acquisition parameters were as follows: Observation System Type: Regular grid, 16 lines, 4 source points, midpoint-triggered. Number of Receiver Lines: 10 per grid line. Total Number of Receiver Channels: 96 × 16 = 1536 channels. Receiver Line Spacing: 40 m. Receiver Channel Spacing: 10 m. Lateral Shotpoint Spacing: 10 m. Vertical Shotpoint Spacing: 60 m. Offset Distance: 10 m. Maximum Lateral Shotpoint Offset: 285 m. Minimum Lateral Shotpoint Offset: 5 m. Maximum Vertical Shotpoint Offset: 480 m. Minimum Vertical Shotpoint Offset: 10 m. Maximum Non-Vertical Shotpoint Offset: 554 m. Common Depth Point (CDP) Grid: 5 m × 5 m. Seismic source points: Fired at a depth 3 m below the water table or at 3 m below the water table when there are artesian wells with an explosive charge of 2 kg. High-sensitivity detectors with a natural frequency of 7 Hz were employed. E-428 high-resolution digital seismometers were used, with a sampling interval of 0.5 ms, a recording length of 2.0 s, and data recorded in SEG-D format. The pre-amplification gain was set at 12 dB, and the full-frequency range was received.

**Figure 3:**
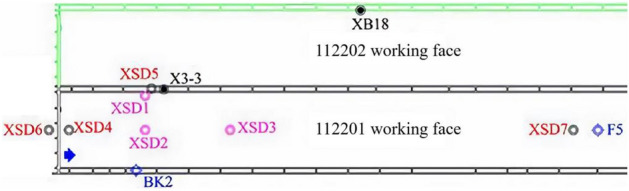
High-density 3D seismic prospecting range.

The high-density 3D seismic detection was carried out near the working face cutting eye, covering an area of approximately 0.31 km^2^ with a lateral-to-vertical ratio of 1:1 within an effective offset distance of 400 m. The detection range spanned from the southwestern boundary, located 60 m outside the working face's cutting eye, to the northeastern boundary, situated 450 m from the cutting eye within the working face. The southeastern and northwestern boundaries were positioned 100 m outside the roadway of the working face (see Fig. 3). This detection range ensured full coverage of high-density 3D seismic data over both unmined and mined coal seams for comparative research. Additionally, within the high-density 3D seismic detection range, exploration boreholes were drilled within the XS-D1, XS-D2, and XS-D4 working faces to investigate water-conducting fracture zones, and two additional boreholes (XS-D5 and XS-D6) were located outside the working faces to compare the characteristics of these fracture zones (see Fig. 3). This approach facilitated a detailed analysis of the spatial variations of water-conducting fracture zones by combining drilling and high-density 3D seismic data. The seismic data acquisition phase commenced right after the drilling project concluded, spanning 14 days. At this point, the 112201 working face had been in operation for ten months, with a total length of 4 km.

#### Analysis of the development and three-dimensional morphology of water-conducting fracture zones

**Figure 4:**
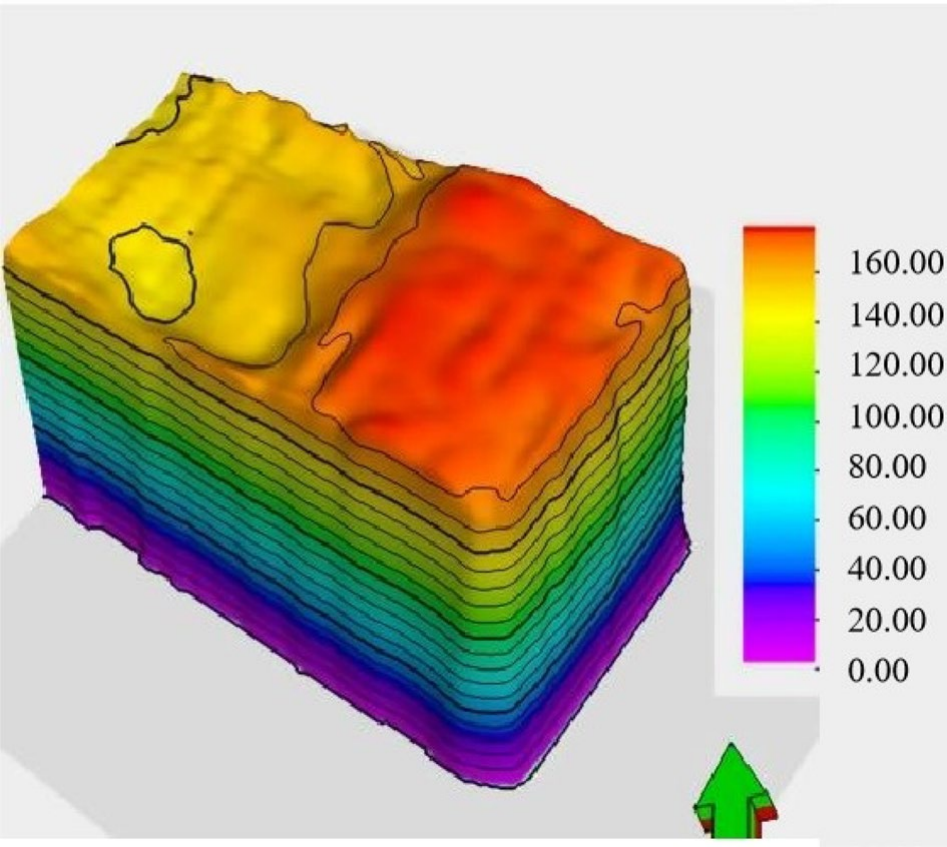
Development height of fracture zone.

As shown in Fig. 4, the fracture zones develop above the goaf, extending approximately 20 m beyond the goaf boundaries. By combining seismic attributes and inversion profile features, it can be determined that the top boundary of these fracture zones, denoted as t0, ranges between 105 to 131 ms. Based on the depth-to-time conversion relationship, the velocity can be calculated as V = − 0.0158t_0_^2^ + 7.8013t_0_ + 1917.7. Consequently, the elevation of the top boundary of the fracture zones falls within the range of 1125–1157 m. In comparison to the roof of the coal seam, the height of development of these fracture zones ranges from 150 to 178.42 m.

**Figure 5:**
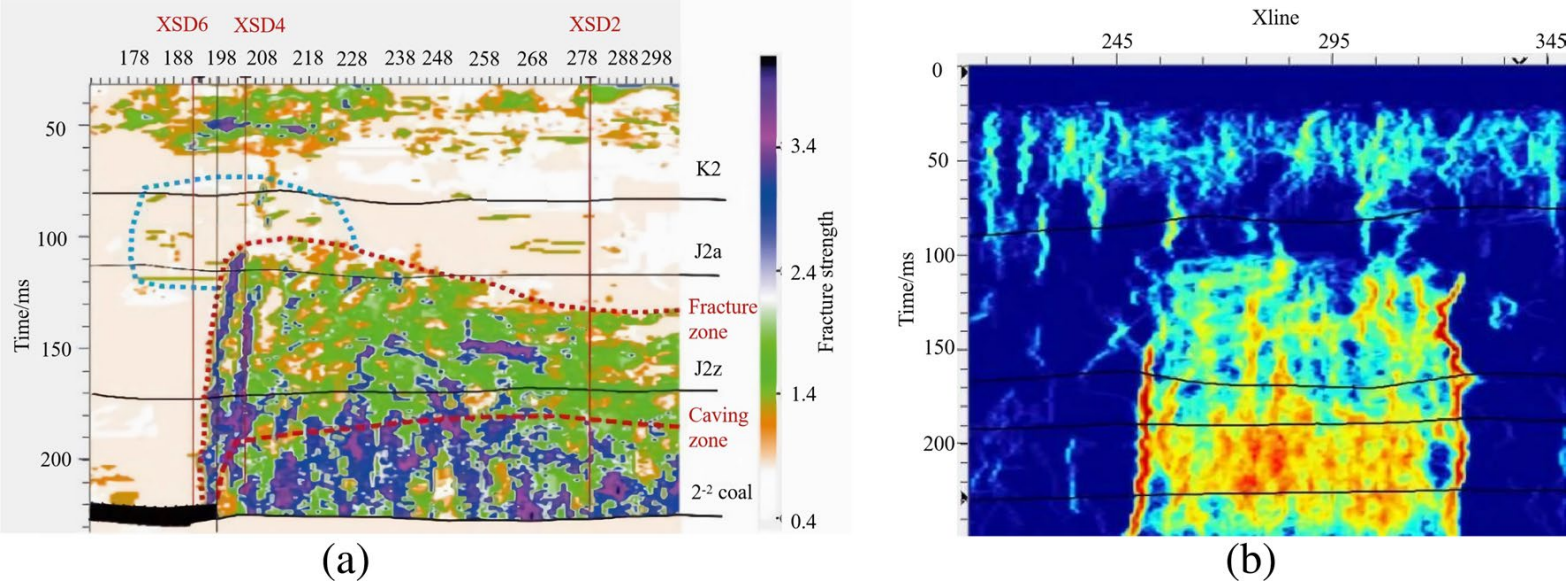
Three-dimensional morphology of water-conducting fracture zone. (**a**) Strike fracture indication inversion, (**b**) dip seismic data fusion.

As shown in Fig. 5 the degree of fracture development increases with depth. These fracture zones can be classified into three zones based on their degree of development, including weakly developed fracture zones, moderately developed fracture zones, and strongly developed fracture zones. It is worth noting that the strongly developed fracture zones exhibit a degree of development close to that of the caving zone. This observation is due to the lack of data concerning the caving zone in this area. In terms of orientation, the development height of the fracture zones follows a north-high, south-low pattern parallel to the working face. In the vertical orientation, the fracture zones show a high-low-high pattern from the sides toward the center. This pattern can be described as a "half-saddle shape."

The fracture development height exhibits a pattern of decreasing height from the goaf towards the mining face and a high-low-high feature from the sides toward the center, both in the strike and dip directions. The southern part of the goaf has the highest fracture development height, reaching approximately 178.42 m. As we move towards the boundaries of the goaf, the development height gradually decreases. In the research area, the coal seam 2-2 has a height of 5.80 m, and the fracture-to-seam ratio ranges from 25.86 to 30.76.

**Figure 6:**
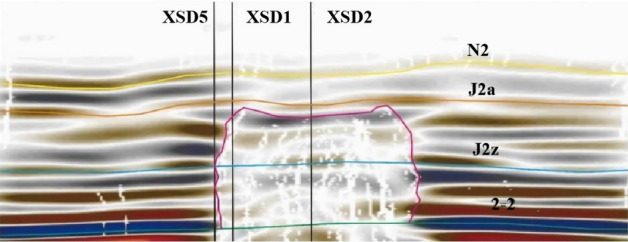
Three-dimensional morphology of water-conducting fracture zone.

As shown in Fig. 6, these fracture zones do not penetrate the bedrock layer and have not reached the weathered bedrock surface. The maximum development height of these fracture zones, as calculated, is 32 m from the bedrock surface. The water-conducting fracture zones do not connect with the water-bearing layers of the Yanchang Formation (Q2l) or the Wucheng Formation (Q1w) of the Quaternary period. These layers serve as confining aquifers between the unconfined water and confined water layers. In this region, the elevation of the bedrock surface is higher than the maximum development height of the fracture zones by 32 m, indicating that the water-conducting fracture zones do not connect with the confining aquifers. The water-conducting fractures have penetrated the Yanchang Formation aquifer and the clastic rock aquifer of the Zhaolu Formation. These aquifers have a relatively weak water-bearing capacity, suggesting that the water-conducting fracture zones are not likely to carry significant water.

**Figure 7:**
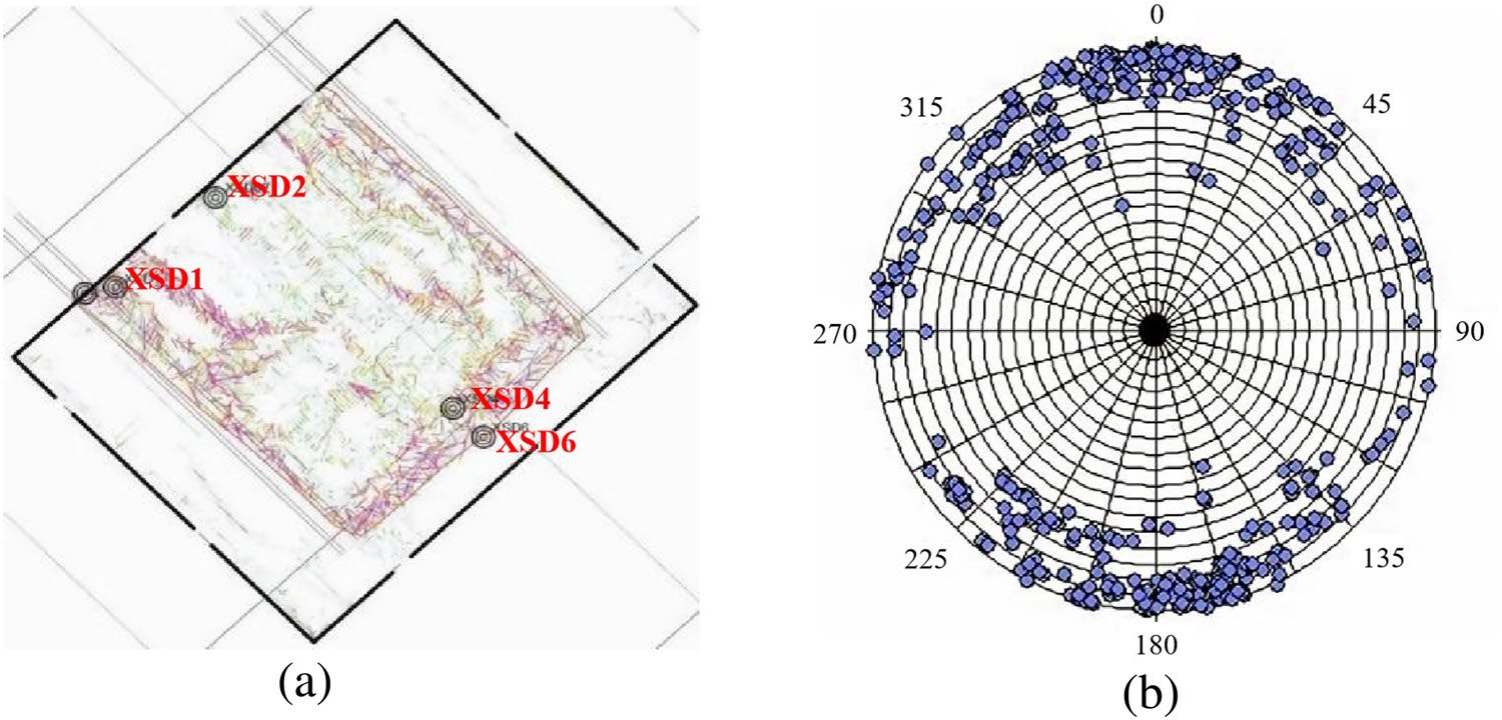
Three-dimensional morphology of water-conducting fracture zone. (**a**) Strike fracture indication inversion, (**b**) dip seismic data fusion.

Figure 7a shows a vector map that combines fracture density and orientation information. This map is created by overlaying data from anisotropy testing, including fracture density data and fracture orientation angle data. In this map, the length of each vector represents the size of a fracture, and the direction of the vector represents the orientation angle of the fracture. As you can see from Fig. 7, fracture density increases gradually with depth. The overall pattern in the water-conducting fracture zone indicates that fracture density is higher at the edges of the working face compared to the central part. The fractures are predominantly high-angle fractures, and their distribution in the horizontal direction appears as a network.

When examining the rock mass around the working face, it's apparent that high-angle and even vertical fractures are prevalent. The fractures are more pronounced on the sides of the working face, while they are relatively weaker in the central part. Within the fracture zone, the number of fractures increases from top to bottom, with high-angle fractures developing in the lower part of the fracture zone and both high-angle and horizontal fractures in the upper part.

By employing an automatic fracture tracking approach on seismic attribute data, over 200 fractures have been interpreted. The rose diagram of fractures is presented in Fig. 7b. It's important to note that the number of tracked fractures does not necessarily represent the actual number of fractures due to factors such as seismic resolution, signal-to-noise ratio, and fracture combinations. However, it does provide an overall indication of the degree of fracture development in the area. The statistical analysis of tracked fractures is a valuable reference for understanding fracture development patterns..

**Figure 8:**
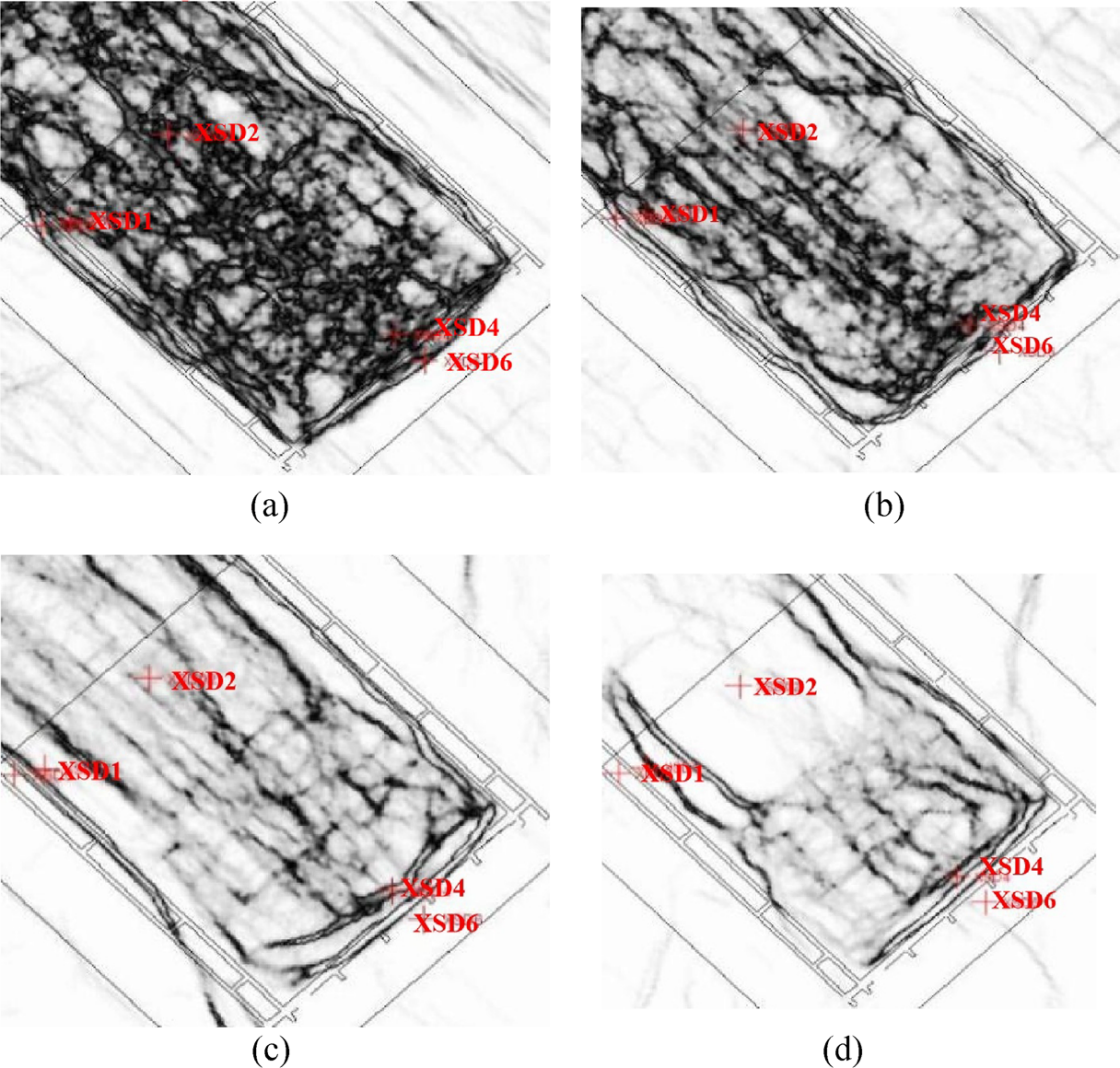
Fracture development in water-conducting fracture zone. (**a**) 200 m caving zone fracture development zone. (**b**) 180 m fracture strong development zone. (**c**) 140 m fracture middle development zone. (**d**) 120 m fracture weakly development zone.

The development degree of fractures continues to strengthen with the increase of buried depth. Therefore, according to the development degree of fractures from top to bottom, we divide the fracture zone into three zones, which are weak fracture development zone, moderate fracture development zone and strong fracture development zone respectively; among them, the fracture development degree of strong fracture development zone is close to that of caving zone in the numerical value of inversion profile, indicating that the fracture development degree of strong fracture development zone is close to that of caving zone, of course, this is also related to the lack of caving zone data in this area. In general, at different depths below the coal seam floor, the development of fractures can be categorized as follows:

In the depth range of 0 to 35 m below the coal seam floor, it is referred to as the "caving zone fracture development zone." In this zone, the intensity of fracture development exceeds 2.5. Moreover, there are some horizontal fractures resembling bedding plane fractures developed at the top of the fracture zone (as shown in Fig. 8a).

Between 35 and 66 m below the coal seam floor, it constitutes the "fracture strong development zone." Here, the intensity of fracture development ranges between 2 and 2.5. These fractures exhibit a densely distributed network pattern throughout the plane (as depicted in Fig. 8b).

From 66 to 124 m below the coal seam floor, there is the "fracture middle development zone." In this zone, the intensity of fracture development falls within the range of 1.4 to 2. The fractures also present a densely distributed network pattern throughout the plane, though the degree of development is somewhat reduced compared to the strong development zone (as illustrated in Fig. 8c).

Between 124 and 178.42 m below the coal seam floor is the "fracture weakly development zone". In this zone, the intensity of fracture development falls within the range of 1–1.4. On the plane, surface fractures near the goaf boundaries further weaken, and the overall density of the network-like distribution becomes less dense (as shown in Fig. 8d).

This comprehensive categorization and analysis provide insights into the variation in fracture development at different depths below the coal seam floor. It is valuable for assessing potential risks and stability in the mining environment.

### Joint well-surface microseismic monitoring for fracture zone height

#### Well-surface microseismic monitoring system and plan

Microseismic monitoring technology has evolved based on seismic monitoring principles and is similar in principle to seismic monitoring and acoustic emission monitoring. It is based on the principles of stress-induced rock failure and the emission of energy. When a coal-rock mass is subjected to stress and reaches a state of imbalance, elastic potential energy accumulates within. When this energy reaches a critical threshold, it triggers fractures within the coal-rock mass, resulting in microseismic events. These microseismic events release energy in the form of seismic waves. These seismic waves are then detected and recorded by sensors and data acquisition devices deployed around the fracture zone. By analyzing the time differences and wave velocities of seismic waves received by different detectors, an equation system can be established to determine the source location of the seismic events. After a large number of microseismic sources have been monitored and located, density analysis and statistical methods can be applied to gain insights into the status of rock mass damage.

The well-surface joint microseismic monitoring technology is a new technique within coal mines, building upon traditional microseismic monitoring. It involves simultaneous deployment of observation points on the surface and underground. The observation points should encircle the monitoring area, enabling comprehensive data collection regarding coal-rock mass fractures. The timing of data collection should be based on the coal seam extraction schedule of the Xiaobaochang coal mine and the equipment used to ensure the acquisition of as much geological data as possible for accurate information on rock layer fractures.

**Figure 9:**
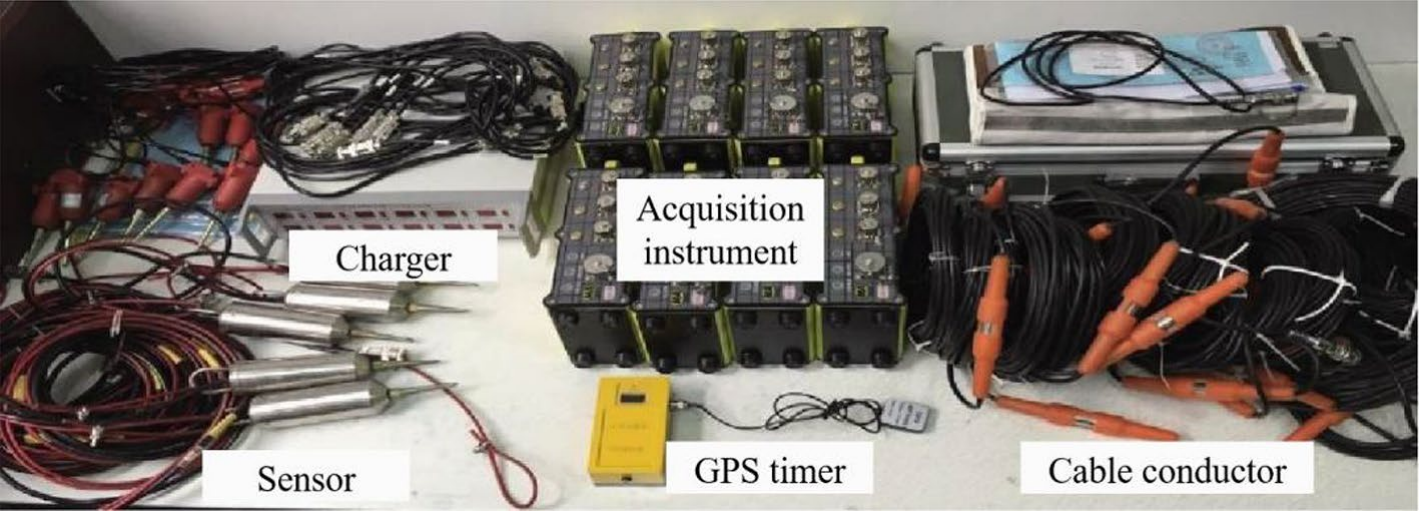
YTZ-3 microseismic monitoring system.

Key components of the microseismic monitoring system include sensors, data acquisition devices, and a central host. The sensors are responsible for identifying and capturing elastic waves generated by coal-rock mass fractures, while the data acquisition devices collect and record the captured microseismic signals. The central host is used for viewing, analyzing, and processing the acquired microseismic signals. The YTZ-3 microseismic monitoring system comprises hardware components such as data acquisition devices, sensors, and cables, as well as software components like configuration software for data acquisition devices, data decoding software, and data processing software. The YTZ-3 microseismic monitoring system is illustrated in Fig. 9.

This well-surface joint microseismic monitoring system is instrumental in understanding the fracture zone height and rock mass behavior during mining operations. It combines surface and underground observations to provide comprehensive insights into the coal-rock mass fracture process.

Following the principles of high-low alternation and uniform distribution of measurement points, and taking into account the actual geological conditions in the monitoring area within the range of 3600–4100 m from the cutting point (first-stage monitoring task), nine microseismic monitoring points were established on the surface. Out of these, five are fixed monitoring points, and the remaining four are mobile monitoring points. Considering the direction of coal face advancement and the layout of monitoring points, XB1-3 and XB1-9 points were designated as mobile monitoring points. When the microseismic signals at these two points weaken, they will be relocated to XB1-7 and XB1-6 points, ensuring that the surface receives reliable microseismic signals throughout the monitoring process.

For the monitoring area 100 m ahead of the stop line (second-stage monitoring task), seven surface monitoring points and five underground monitoring points were established. In this case, due to the relatively short monitoring distance, both the surface and underground monitoring points are fixed monitoring points.

#### Height analysis of water-conducting fracture zone

The monitoring was conducted in two phases. The first phase commenced on August 25, 2019, and concluded on October 7, covering a monitoring distance of 510 m and spanning 44 days. The second phase began in the afternoon of November 17, 2019, and ended on December 11, encompassing a shorter monitoring distance of 120 m. In total, the monitoring covered a distance of 530 m and successfully located 8058 microseismic events.

**Figure 10:**
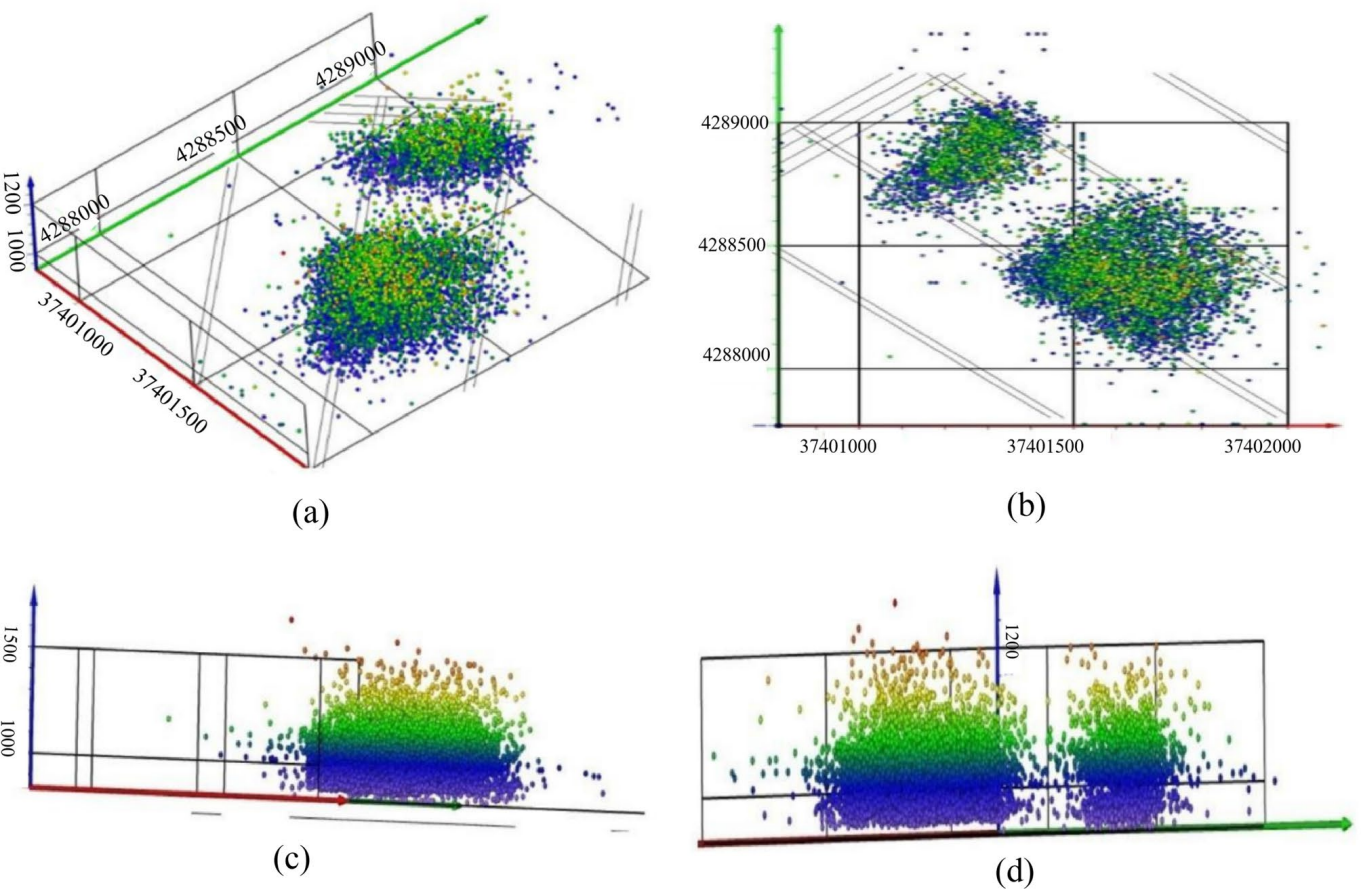
Spatial distribution characteristics of microseismic events. (**a**) Three-dimensional spatial distribution map of microseismic events. (**b**) XY plane distribution map of microseismic events. (**c**) Distribution map of microseismic events along the working face. (**d**) Distribution map of microseismic events along the working face.

**Figure 11:**
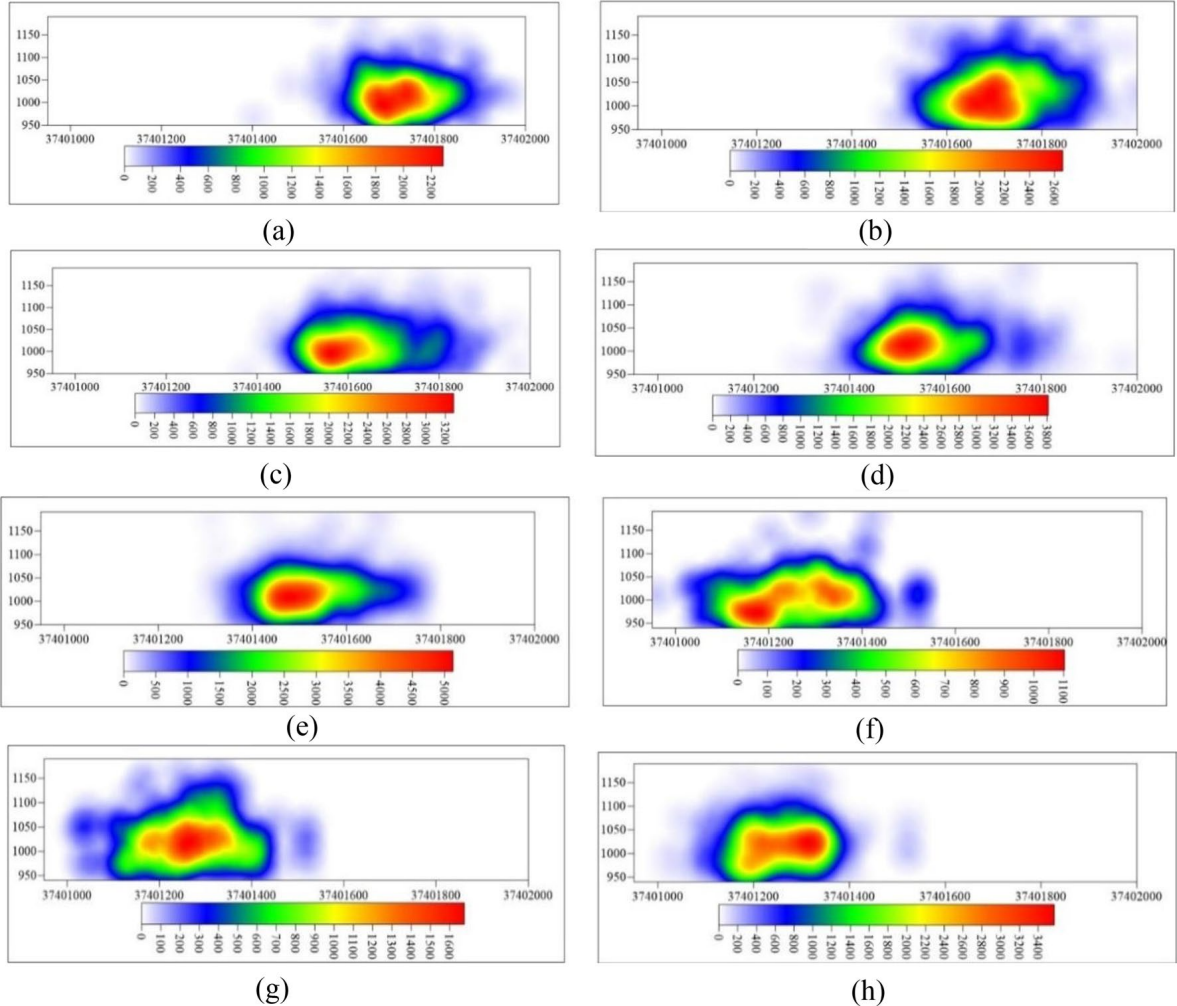
YZ plane microseismic event density nephogram. (**a**) 8.25–8.31, (**b**) 9.1–9.8, (**c**) 9.9–9.19, (**d**) 9.20–9.27, (**e**) 9.28–10.7, (**f**) 11.17–11.2, (**g**) 11.26–12.6, (**h**) 12.7–12.11.

As shown in Fig. 10, we have the three-dimensional spatial distribution of microseismic events during the monitoring period (Fig. 10a), the XY-plane distribution of microseismic events, microseismic events distribution along the inclination of the working face, and microseismic events distribution along the direction of the working face. When looking at the XY-plane map, we can observe that the rupture points are mainly concentrated within the monitoring area of the 112201 working face, and their overall orientation is perpendicular to the direction of the working face (Fig. 10b). Examining the distribution along the inclination of the 112201 working face, we notice that the microseismic events along the sides of the working face tend to have slightly higher development heights compared to those within the working face (Fig. 10c). Additionally, apart from receiving rupture signals from within the 112201 working face, we also received rupture signals from the exterior of the working face. As seen in Fig. 11d, rupture points predominantly occur within the elevation range of 940–1160m.

The total monitoring duration of microseismic activity was 56 days, with a total monitoring distance of 1040 m. The microseismic data was divided into approximately 7-day intervals corresponding to 8 stages, starting from the beginning of the monitoring period to the end of the monitoring period (8.25–8.31, 9.1–9.8, 9.9–9.19, 9.20–9.27, 9.28–10.7, 11.17–11.25, 11.26–12.2, 12.3–12.11).

Figure 11 displays the microseismic event density cloud map in the XZ (east–west direction) plane. The density of microseismic events in the first monitoring phase is greater than that in the second monitoring phase. This is because the 112201 working face had lower mining intensity towards the end of the mining process, and artificial reinforcement was applied to the end of the 112201 working face due to caving issues in the roof during mining. By the time the second monitoring took place, rock strata above the roof of the coal seam at the end of the 112201 working face had already experienced fracturing, resulting in a lower density of microseismic events in the second monitoring phase. Using a microseismic event density value of 600 as the standard for high-density microseismic event areas, the elevation boundaries of the high-density microseismic event areas from the beginning of monitoring to the end of monitoring in each time period are + 1087 m, + 1118 m, + 1112 m, + 1125 m, + 1111 m, + 1062 m, + 1105 m, and + 1123 m, respectively. It is evident that during the monitoring period, the elevation of the upper boundary of high-density microseismic event areas above the 112201 working face reached its maximum at + 1125 m.

**Figure 12:**
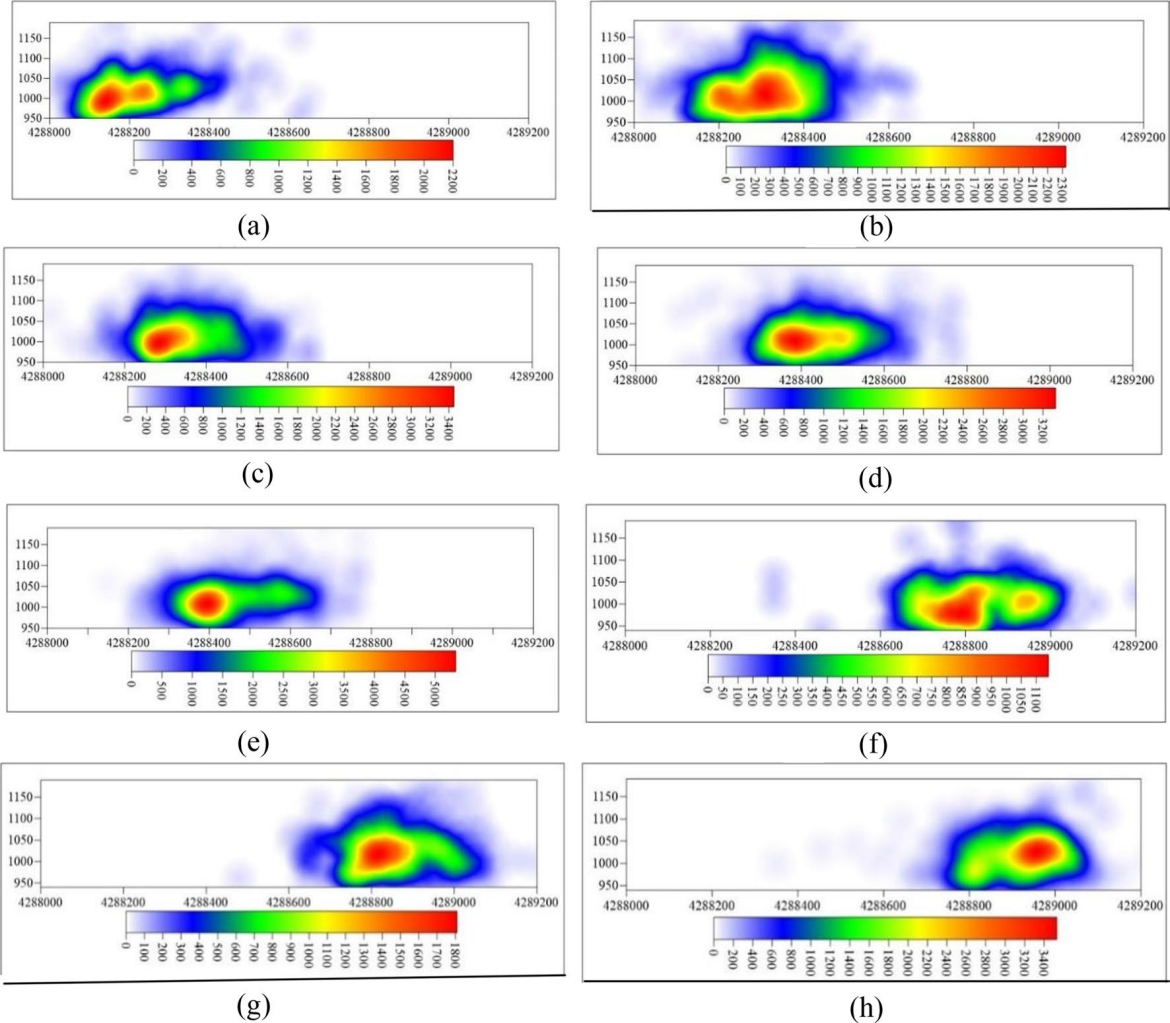
YZ plane microseismic event density nephogram. (**a**) 8.25–8.31, (**b**) 9.1–9.8, (**c**) 9.9–9.19, (**d**) 9.20–9.27, (**e**) 9.28–10.7, (**f**) 11.17–11.2, (**g**) 11.26–12.6, (**h**) 12.7–12.11.

Figure 12 presents the microseismic event density cloud map in the YZ plane. The elevation boundaries of high-density microseismic event areas, as well as the density values, gradually increase with monitoring time. Using a microseismic event density value of 600 as the standard for high-density microseismic event areas, the elevation boundaries of the high-density microseismic event areas from the beginning of monitoring to the end of monitoring in each time period are + 1080 m, + 1127 m, + 1101 m, + 1106 m, + 1099 m, + 1044 m, + 1083 m, and + 1104 m, respectively. It is apparent that, except for the two initial monitoring time intervals where the elevation of high-density areas was relatively low, the elevation of high-density boundary areas in the remaining time intervals ranged from + 1093 to + 1128 m. During the monitoring period, the high-density microseismic event areas could develop up to a maximum elevation of + 1128 m.

**Figure 13:**
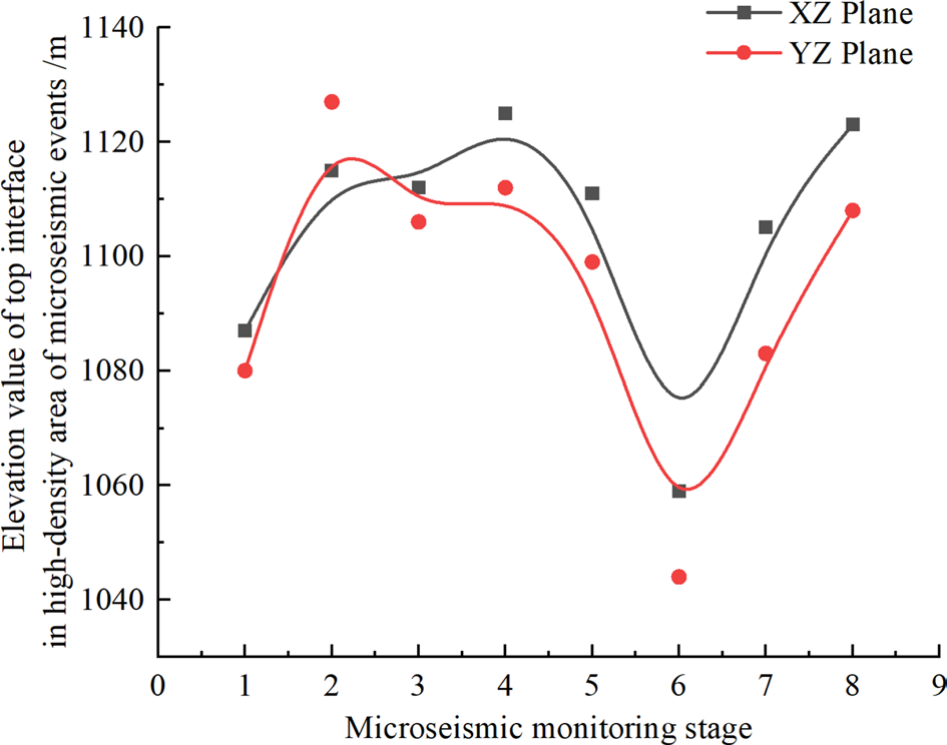
Comparison diagram of top interface in high-density area of XZ-YZ microseismic event.

In this microseismic monitoring project, the height of the water-conducting fracture zone was determined using a combination of density analysis and the vertical distribution characteristics of microseismic events in different time periods. As seen in Fig. 13, both in the XZ and YZ planes, during the initial monitoring stages (8.25–8.31, 11.17–11.25, 11.26–12.2), the elevation of the upper boundary of the high-density microseismic event areas was relatively low. This is because the fracturing of the overlying strata was in an advanced stage before monitoring. The roof had already fractured in the monitoring area before the monitoring took place, but the microseismic signals from these fractures were not received, resulting in fewer microseismic events during the initial monitoring stages. In the later stages of monitoring (9.1–9.8, 9.9–9.19, 9.20–9.27, 9.28–10.7, 12.3–12.11), the elevation boundaries of high-density microseismic event areas in the XZ and YZ planes varied significantly, with the XZ plane generally having higher elevation boundaries than the YZ plane.

Taking into account the vertical changes of microseismic events in the strata during different monitoring time periods, microseismic events gradually increased as the working face was mined. In the initial monitoring stages of the first and second phases, the number of microseismic events at various elevations was relatively low compared to the later stages of monitoring. This was due to the disturbance of the overlying strata stress equilibrium in front of the working face during mining, leading to increased stress in the overlying strata and advanced fracturing of the overlying strata ahead of the working face, resulting in fewer microseismic events during the initial monitoring stages.

**Figure 14:**
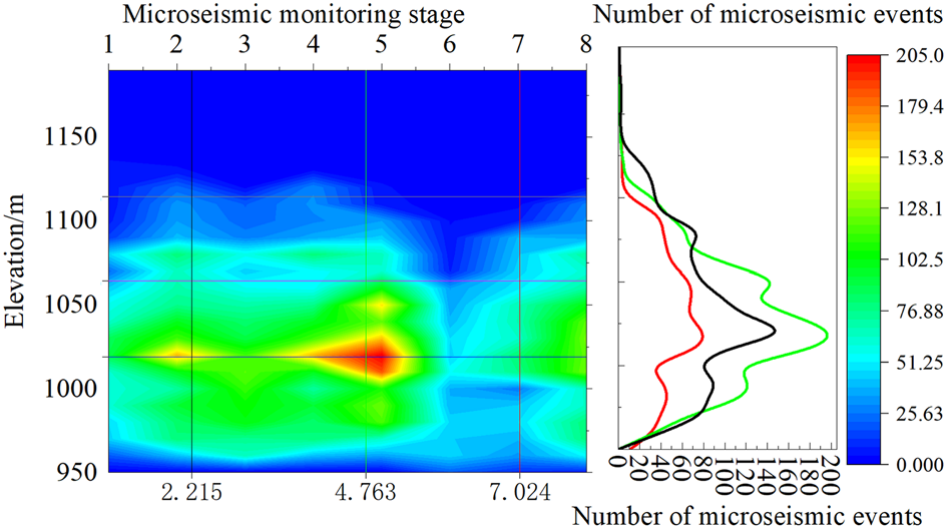
Vertical variation of microseismic events in each monitoring period.

Figure 14 reveals that during the time intervals of 9.1–9.8, 9.9–9.19, 9.20–9.27, 9.28–10.7, and 12.3–12.11, the number of microseismic events vertically decreased rapidly at elevations above + 1110  to  + 1120 m, + 1100 to  + 1110 m, + 1110 to  + 1120 m, + 1090 to  + 1100 m, + 1100 to  + 1110 m, respectively. By comparing the elevation values of high-density areas in the XZ and YZ planes, it is evident that the elevation values of the water-conducting fracture zone during normal monitoring periods were + 1115 m, + 1106 m, + 1112 m, + 1108 m (9.1–9.8, 9.9–9.19, 9.20–9.27, 9.28–10.7, 12.3–12.11), indicating that the height of the water-conducting fracture zone was 163m, 154m, 160m, 168m, respectively.

From the distribution pattern of microseismic events and their relationship with the water-conducting fracture zone, it can be observed that microseismic events are primarily distributed in the area where the water-conducting fracture zone intersects with the bending subsidence zone. Since the rock mass within the water-conducting fracture zone contains numerous interconnected fractures, it indicates that the rock mass within the water-conducting fracture zone is primarily in a state of fracturing. As microseismic events increase, the rock mass gradually transitions from a stable state to an unstable state, indicating poorer stability of rock specimens, approaching the state of fracturing.

In summary, the height of the water-conducting fracture zone near the goaf is slightly greater than within the working face, which is consistent with the theoretical research on water-conducting fracture zones. However, it must be noted that during the monitoring process of the 112201 working face, artificial reinforcement was applied to the roof near the goaf due to roof caving issues. Additionally, the coal seam had lower mining intensity at that time. After the closure of this working face, the water-conducting fracture zone is expected to develop further upwards. With the conclusions obtained so far, it can be determined that the height of the water-conducting fracture zone within the 112201 working face ranges from 154 to 163m, with a fracture extraction ratio of 26.55 to 28.10. Near the goaf at the stopping line, the height of the water-conducting fracture zone is 168m, with a fracture extraction ratio of 28.97.

## Experimental verification

### Numerical simulation

#### Experimental design for numerical simulation

Based on the K4-3 borehole composite column diagram and the rock mechanics parameters table (Table 1), a numerical simulation model was constructed. The numerical simulation was designed for longitudinal mining, with each step advancing by 50 m, divided into 14 steps. The upper coal seam (2-2 coal) was mined first, followed by the lower coal seam (3-1 coal) after completing the upper seam mining. During the mining process, vertical cross-sections were analyzed along the direction to examine the extent of the plastic zone and determine the development range and height of the three zones. Considering the layout of the working face, 50 m-wide coal pillars were preserved on both sides, and 50 m was preserved in the front and rear. The model dimensions were set to be 800 m × 350 m × 365.7 m, with an inclination angle of 1°. The grid cells were 10 m × 10 m × 2 m, resulting in a total of 152,000 grid cells.

#### Distribution characteristics of the plastic zone during the mining of the 2-2 coal seam

**Figure 15:**
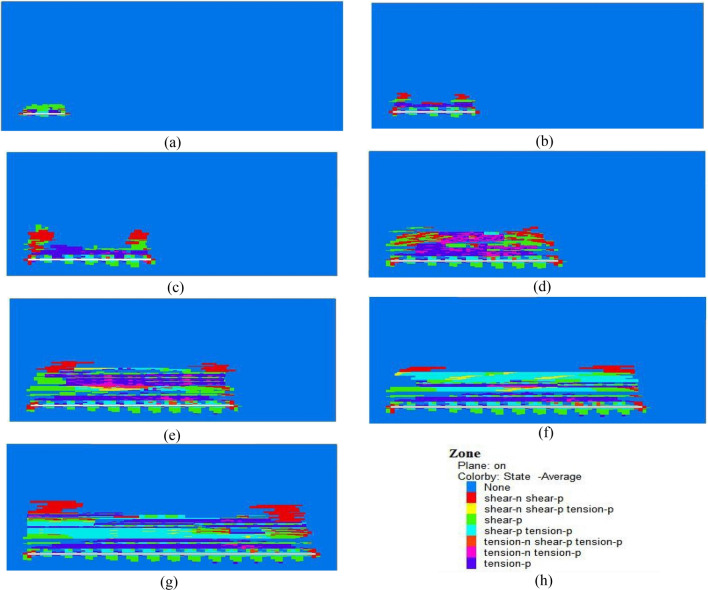
Slices of the plastic zone (cut along the XZ plane). (**a**) Plastic zone at 100 m, (**b**) plastic zone at 200 m, (**c**) plastic zone at 300 m, (**d**) plastic zone at 400 m, (**e**) plastic zone at 500 m, (**f**) plastic zone at 600 m, (**g**) plastic zone at 700 m, (**h**) example of a plastic zone diagram.

As shown in Fig. 15, the simulation of the plastic zone during the excavation of the 2-2 coal seam resulted in cross-sectional profiles of the 2-2 coal seam and its overlying rock along the XZ plane.

During the underground mining of coal mines, the natural stress equilibrium of the overlying rock mass is disrupted as the underground working face advances. As a result, secondary stress fields are redistributed, causing internal stress within the rock layers to redistribute to achieve a new equilibrium. This leads to fractures, caving, bending, and subsidence in the overlying rock layers. These changes in movement progressively propagate upwards, eventually causing surface subsidence.

When the working face advanced to 100 m, changes in the plastic zone were observed. The overlying rock mass underwent tension and shear stress, and the plastic zone height developed up to 22 m (Fig. 15a). At 200 m of face advancement, the influence of the plastic zone expanded, and there was notable tension and shear stress in the overlying rock mass. The shear damage occurred at the coal wall and cutting locations, forming an approximate "saddle" shape. The plastic zone height reached 54.28 m (Fig. 15b). At 300 m of face advancement, the influence of the plastic zone further expanded, with symmetrical development on both sides. The plastic zone height increased to 94.7 m (Fig. 15c). At 400 m of face advancement, the plastic zone's influence continued to grow, and tension damage increased in the overlying rock mass above the goaf. Multiple layers of rock showed tension damage. The development of the plastic zone height reached 106.8 m and exhibited a pronounced saddle-like shape (Fig. 15d). At 500m of face advancement, the tension effect above the goaf was significant, and the plastic zone height increased to 117.69 m, exhibiting a symmetrical development (Fig. 15e). At 600 m of face advancement, the influence of the plastic zone continued to expand, with slight increases in the plastic zone height on both sides. The overlying rock experienced a tension effect, and the plastic zone height reached 138.6m (Fig. 15f). At 700 m of face advancement, the influence of the plastic zone further expanded, and the profile exhibited a symmetrical "saddle" shape. The plastic zone height reached 169.2 m and stabilized thereafter (Fig. 15g).

### Similar material simulation analysis

#### Similar material simulation analysis

**Figure 16:**
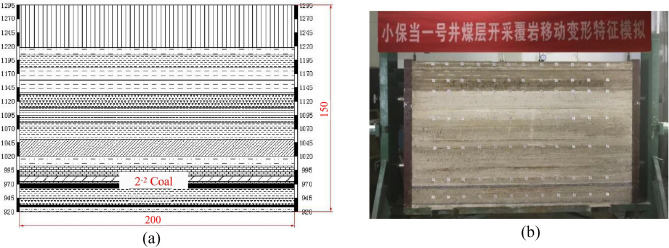
Similar material simulation experiment. (**a**) Cross-section of simulated geological layers. (**b**) Model diagram for similar material simulation experiment.

In this experiment, Xiaobaodang Coal Mine No. 1 well, working face 112201, was taken as a prototype. Based on the objectives of the similar material simulation experiment and a thorough analysis of geological data in the study area, the geological layers for the simulation were determined, as shown in Table 1. The equipment used for the similar material simulation experiment had dimensions of 2.0 m in length, 1.5 m in height, and 0.2 m in width, with a scale ratio of 1: 250. The simulated geological strata had a total thickness of approximately 375 m, with two coal seams designated for mining: the 2-2 coal seam at a depth of 320 m and the 3-1 coal seam at a depth of 365 m. Both coal seams were horizontally layered, with thicknesses of 6.25 m and 3.75 m, respectively. The roof rock was composed of siltstone, and the floor rock was also siltstone. The mining speed for each coal layer was set at 25 m per step (equivalent to 10 cm per step). There were 20 cm coal pillars left on both sides of the model, and the mining area for the 2-2 and 3-1 coal seams was 170 cm long, divided into 17 steps, with each step being 10 cm. For the experimental model and dimensions, please refer to Fig. 16 and Table 2.

**Table 2:** Model Dimensions and Mining Conditions.

Category	Horizontal length	Vertical height	Overlying rock thickness	Coal pillar width	Coal seam height
Prototype size/m	500	375	320/365	20	6.25/3.75
Model size/cm	200	150	128/146	20	3/1.5

#### Development patterns of water-bearing fracture zones

When mining the 2-2 coal seam, the heights of caving zones obtained from the experiment changed as the mining face advanced. At a mining face advancement of 50 cm, the goaf area experienced initial collapse, with a step distance of 43 cm, a collapse zone height of 2 cm, a collapse angle of 65° on the cutting side, and 70° on the working face side. When the mining face advanced to 60 cm, the goaf area experienced a second collapse, with a step distance of 10 cm, a collapse zone height of 5cm, a collapse angle of 65° on the cutting side, and 68° on the working face side. At a mining face advancement of 150 cm, the goaf area experienced the fifth collapse, with a step distance of 11cm, a collapse zone height of 21 cm, a collapse angle of 65° on the cutting side, and 58° on the working face side. Subsequently, the development height of the collapse zone remained relatively stable and did not continue to rise as the mining face advanced. The overlying rock collapse remained stable on the cutting side, while on the working face side, the collapse angle continued to change with coal seam excavation. In summary, when mining the 2-2 coal seam, the initial collapse step distance was 43 cm, and the average overburden step distance at other locations was 10.58 cm. Hanging walls collapse with each subsequent mining step. The maximum height of caving zones created by mining the 2-2 coal seam was 19 cm, which corresponds to a depth of 47.5 m in reality.

**Figure 17:**
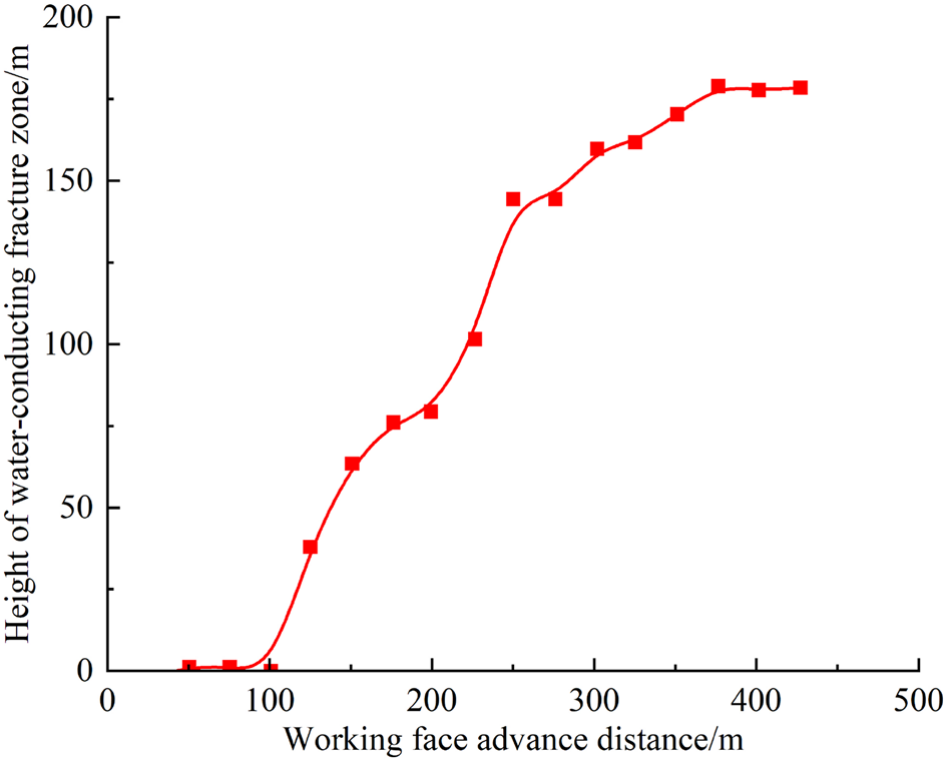
Development patterns of water-bearing fracture zones.

The development pattern of water-bearing fracture zones obtained from the similar material simulation experiment as the mining face advanced is shown in Fig. 17. It is evident that the change in water-bearing fracture zone height exhibits a trapezoidal increase pattern. After mining the 2-2 coal seam, the upper overlying rock first experienced a break at a mining face advancement distance of 125 m. As the mining face continued to advance, the height of the water-bearing fracture zone increased, reaching around 177.5 m at a mining face advancement distance of 425 m. In the initial stages of the 2-2 coal seam mining, the height of overlying rock damage essentially formed a horizontal straight line, with a relatively small damage range. When the mining face advanced to 100 m, the height of overlying rock damage rapidly increased. As the advancement distance reached 300 m, the rate of damage height growth slowed down, and by the time the advancement distance reached 425 m, it had reached 177.5 m. The primary reason for this development pattern is the periodic breaking and deformation of the overlying rock layers, resulting in an overall stepped curve pattern.

#### Determination of height of water-conducting fracture zone

This project investigated the development height of water-bearing fracture zones in the Xiaobaodang No. 1 well test mining area using five different methods: similar material simulation, numerical simulation, borehole exploration, 3D seismic exploration, and joint well-geomechanical microseismic monitoring.

1. Based on Eqs. (3) and (11), the estimated height of the upward water-conducting fracture zone is 170.10 m (28 times the mining height), and the predicted depth of the downward water-conducting fracture for single coal seam mining is 2.75 m.
2. Similar Material Simulation: The study found that during the mining of the 2-2 coal seam in the research area, the fracture zone exhibited a stepped curve pattern, mainly influenced by periodic breaking and deformation of overlying rock layers. At a mining face advancement of 425 m, the height of the fracture zone reached 177.5 m.
3. Numerical Simulation (FLAC3D): When the 2-2 coal seam in the research area is mined separately, the "plastic zone" method yielded a water-bearing fracture zone height of 169.2m.
4. Borehole Exploration ("Three-Zone" Drilling): Comprehensive results from borehole exploration, including simple hydrological observations, geological core recording, conventional geophysical logging, and television logging, indicated that the height of water-bearing fracture zones in the Xiaobaodang No. 1 coal mine's 112201 working face goaf area ranged from 152.013m to 175.573 m, with a fracture ratio of 26.21 to 30.27.
5. High-Density 3D Seismic Exploration: The results from 3D seismic exploration showed that the development boundary of water-bearing fracture zones extended approximately 20 m beyond the goaf boundary. The height of development of these zones ranged from 150 to 178.42 m, with a fracture ratio between 25.86 and 30.76. The fractures were predominantly high-angle, oriented along the vertical and parallel directions to the working face.
6. Joint Well-Geomechanical Microseismic Monitoring: This monitoring, based on statistical principles and the distribution density of microseismic events, determined that at a mining height of 5.8m, the development height of water-bearing fracture zones within the working face ranged from 154 to 163m, with a fracture ratio between 26.55 and 28.10. Near the stop line, the height of water-bearing fracture zones was found to be 168m, with a fracture ratio of 28.97.

In a comprehensive analysis of these results, it was observed that different methods produced slightly varying maximum development heights of water-bearing fracture zones, which generally fell within the range of 163–178 m. The fracture ratio within the test mining area ranged from 25.86 to 30.76.

## Conclusion

1. Calculations based on the critical stratum theory for the height of water-bearing fracture zones indicate that when a single layer of the 2-2 coal seam is mined, the projected height of water-bearing fractures is 170.10m, and the main critical stratum remains undamaged. When mining the 2-2 single coal seam downward, the expected depth of water-bearing fractures is 2.75 m, revealing the influence of the overlying stratum's critical layer on the development height of water-bearing fractures. When the maximum deflection of the critical layer is less than the maximum free space below the critical layer, rock strata undergo damage, and water-bearing fracture zones continue to develop upwards. A method for elastic wave exploration of the height of water-bearing fracture zones based on the critical stratum theory is proposed.
2. High-density 3D seismic survey results based on the critical stratum theory indicate that in the mining of the 2-2 coal seam, the development boundary of fracture zones extends about 20 m beyond the goaf boundary. The height of development of water-bearing fracture zones ranges from 150 to 178.42 m, with a fracture ratio between 25.86 and 30.76, and the maximum development height is near the roadway. The 3D morphology of the roof water-bearing fracture zones in the Yushen mining area has been obtained. The morphology of fracture zone development height exhibits characteristics of decreasing height from the goaf towards the working face, as well as decreasing height from both sides along the goaf towards the interior of the working face. It also displays saddle-shaped features in both the strike and dip directions.
3. Joint well-geomechanical monitoring results based on the critical stratum theory show that the development height of water-bearing fracture zones during the mining of the 2-2 coal seam ranges from 154.00 to 168.00 m, with a fracture ratio between 26.55 and 28.97. Inside the working face, the maximum development height of water-bearing fracture zones is 163 m, with a maximum fracture ratio of 28.10; near the stop line, the height of water-bearing fracture zones is 168 m, with a fracture ratio of 28.97. The primary characteristics of rock fracture distribution during mining are that fractures mainly develop along high-angle or even vertical strata layers. Within the fracture zone, fractures increase from top to bottom, with high-angle fractures developing in the lower part of the fracture zone, and high-angle and horizontal fractures developing in the upper part of the fracture zone.
4. The development of water-bearing fracture zones follows a process from formation to development, reaching their maximum height and stabilizing as the coal seam is mined, overlying strata settle, and strata are damaged. Similar material simulation results indicate that after full mining of the 2-2 coal seam, the maximum development height of water-bearing fracture zones is approximately 177.5 m, with a fracture ratio of 28.4. Numerical simulation results show that after full mining of the 2-2 coal seam, the development heights of water-bearing fracture zones determined by the plastic zone method is 169.2 m, with fracture ratios of 29.27. When comparing the results obtained from the elastic wave exploration method based on the critical stratum theory with those from borehole observation, similar simulation, and numerical simulation, they are generally consistent.

## Data Availability

The data used to support the findings of this study are available from the corresponding author upon request.
